# Cross-Contamination of Foodborne Pathogens During Juice Processing

**DOI:** 10.3390/biology14080932

**Published:** 2025-07-24

**Authors:** Isma Neggazi, Pilar Colás-Medà, Inmaculada Viñas, Isabel Alegre

**Affiliations:** Postharvest Biology and Technology Unit, Department of Food Technology, Engineering and Science, University of Lleida, AGROTECNIO-CERCA Center, Av. Rovira Roure 191, 25198 Lleida, Spain; isma.neggazi@udl.cat (I.N.); pilar.colas@udl.cat (P.C.-M.); inmaculada.vinas@udl.cat (I.V.)

**Keywords:** surface transfer, fruit, vegetable, foodborne pathogens, unpasteurized juice, juice processing, hygiene practices

## Abstract

The demand for unpasteurized fruit juices has grown due to their natural nutritional benefits, but this also increases the risk of foodborne illnesses. This study evaluated the transfer of three pathogens (*Salmonella enterica*, *Escherichia coli* O157:H7, and *Listeria monocytogenes*) from different surfaces (cutting boards, knives, and gloves) to produce and subsequently across different juice batches. Cutting boards and gloves showed the highest pathogen transfer rates (ranging from 2.03 ± 4.36 to 70.69 ± 23.58% for cutting boards, and from 0.04 ± 0.05 to 70.61 ± 23.51% for gloves), while knives exhibited the lowest (from 1.27 ± 1.35 to 7.87 ± 5.33%), when surface-to-produce transference was evaluated. Among the tested produce, beetroot had the highest pathogen transfer for all the tested pathogens (for the cutting board, from 48.55 ± 21.66 to 70.69 ± 23.58%, for the knife from 7.17 ± 6.17 to 7.87 ± 5.33%, and for the gloves from 48.85 ± 21.66 to 70.61 ± 23.51%). Beetroot juice provided the most favorable conditions for bacterial transfer (δ = 0.53–0.56; *k*_max1_ = 3.09–3.20), whereas strawberry juice led to the fastest microbial decrease (δ = 1.10–1.26; *k*_max1_ = 2.08–2.28) throughout processed juices. Apple juice demonstrated intermediate bacterial decline rates (δ = 0.75–1.10; *k*_max1_ = 2.20–2.61). These findings highlight the need for improved hygiene practices and contamination control in juice processing to minimize food safety risks associated with unpasteurized fruit or vegetable juices.

## Simple Summary

The increasing popularity of unpasteurized fruit juices raises concerns about food safety, as harmful bacteria can transfer from contaminated surfaces to fruits and vegetables and persist in juice. This study examined how three pathogens (*Salmonella enterica*, *Escherichia coli* O157:H7, and *Listeria monocytogenes*) transfer from surfaces such as cutting boards, knives, and gloves to fresh produce and later into juice. The results showed that cutting boards and gloves facilitated the highest bacterial transfer, while knives posed a lower risk. Pathogen survival in juice varied, with strawberry juice being the matrix with the slowest bacterial decline and beetroot showing the fastest bacterial decline. Apple juice exhibited intermediate effects. These findings suggest that unpasteurized juice manufacturers should be aware of contamination risks during processing, especially when there is a lack of thermal treatment. Proper hygiene practices, surface material choices, and awareness of fruit or vegetable properties can help reduce health risks associated with fresh juice consumption.

## 1. Introduction

The growing demand for fresher and healthier fruit juices has greatly influenced the current production methods, emphasizing minimal processing to preserve heat-sensitive nutrients [1]. As a result, self-service and freshly prepared juices with shelf lives under 48 h have become popular. However, inadequate production practices, especially when pasteurization or high-pressure processing are avoided, pose a substantial risk of pathogen transmission during juice processing [1,2]. Unpasteurized juices, particularly those combining fruits and vegetables such as tubers, carry elevated microbiological risks due to higher initial microbial loads and higher pH levels, both of which can promote pathogen survival [1]. Moreover, cross-contamination from raw ingredients, equipment, and surfaces (e.g., knives, cutting boards, and gloves) can transfer foodborne pathogens, emphasizing the need for stringent hygiene practices during production [2,3,4].

Several outbreaks of foodborne illness linked to unpasteurized juices have been documented, involving pathogens such as *Salmonella* spp. and *Escherichia coli* O157:H7 in orange juice, apple cider, and apple juice [5,6,7,8]. These microorganisms are particularly concerning due to their ability to persist in low-pH environments, such as fruit juices [9]. Although *Listeria monocytogenes* has been less frequently associated with outbreaks in unpasteurized juices, it still constitutes a significant risk for vulnerable populations, given reports of its survival at pH values as low as 3.75 [10,11].

Pathogen contamination of fresh produce can occur at multiple stages, from preharvest exposure to contaminated water, insects, or manure, to postharvest handling, transportation, and storage [3,12,13,14]. Once present, microorganisms may adhere to produce surfaces or penetrate internal tissues, making them resistant to standard washing. For instance, *E. coli* 0157:H7 has demonstrated the ability to adhere to plant surfaces and, in some cases, internalize within plant tissues, evading conventional washing processes [15]. Consequently, any contamination on the product may be transferred to various processing utensils, including cutting boards, knives, and gloves [16,17]. These transfer events can depend on numerous factors such as the equipment’s material and design, environmental conditions, and the matrices involved [3,12].

Although research on the transfer of pathogens from fruits and vegetables remains limited, recent studies have addressed key aspects of this issue. For example, Jung et al. [18] examined *Salmonella* transfer from contaminated citrus peels to both the edible portion and gloves during manual peeling, reporting variability in transfer rates (0.16–5.41%) depending on the citrus type and inoculation site. Additionally, *Salmonella* transfer from gloves to oranges was observed to vary from 0.79 to 8.97%, for navel oranges, and from 0.41 to 1.35% for grapefruit. In another study, Qi et al. [19] investigated the influence of glove materials, pressing conditions, and contact times on *L. monocytogenes* transfer to fresh cantaloupe. Although the results did not show significant effects of these factors, *L. monocytogenes* persisted on gloves even after 85 consecutive contacts, highlighting the potential for long-term contamination of gloves through repeated handling. Furthermore, studies on pathogen persistence have shown that once microorganisms are introduced into fresh juices, their survival can change significantly depending on the juice matrix. For instance, *Salmonella* and *E. coli* O157:H7 have demonstrated prolonged survival in juices with higher pH levels and buffering capacities, such as in beetroot juice [20], while acidic juices, like strawberry juice, promote faster bacterial decline due to the presence of organic acids and polyphenols [21,22]. However, these pathogens possess acid-adaptive mechanisms, allowing them to persist under certain conditions. *L. monocytogenes*, though generally more sensitive to acidity, can survive in juices containing bioactive compounds that mitigate acid stress, further increasing the risk of persistence in juice products [23].

In view of these findings and the continuing need for further investigation, this study aims to examine the dynamics of pathogen transfer throughout fresh juice production. Specifically, it assesses the transfer of *S. enterica*, *E. coli* O157:H7, and *L. monocytogenes* from contaminated surfaces (cutting boards, knives, and gloves) to fruits (strawberry and apple) and vegetables (beetroot), and subsequently into juice, in order to assess their potential for cross-contamination during juice processing. Additionally, it evaluates the transfer of these pathogens from contaminated produce through six consecutive juice batches elaborated with non-contaminated produce. By understanding the transfer dynamics of these pathogens, this study provides valuable insights into food safety risk assessment in unpasteurized juice production.

## 2. Materials and Methods

### 2.1. Preparation of the Bacterial Suspensions

A cocktail of *S. enterica*, *E. coli* O157:H7, and *L. monocytogenes* was used in these experiments. For *S. enterica*, *Salmonella enterica* subsp. *enterica* Monteviedo (ATCC BAA 710), Gaminara (ATCC BAA 711), and Enteritidis (CECT 4300) were used. A single strain of *E. coli* O157:H7, corresponding to ATCC 700728, was used, and for *L. monocytogenes*, CECT 940 (serovar 4d), CECT 4031 (serovar 1a), and CECT 4032 (serovar 4b) were used.

To prepare the cocktail, a single colony was taken to perform a triple streak for each bacterium. For *S. enterica* and *E. coli* O157:H7 strains, the triple streak was performed onto Trypto-casein Soy Agar (TSA, Biokar, France), and for *L. monocytogenes*, in TSA enriched with 6 g/L of yeast extract (Biokar, Allonne, France) (TSAYE). Plates were incubated at 37 °C for 24 h. Then, a single colony from each plate was taken to inoculate 50 mL of Trypto-casein Soy Broth (TSB, Biokar, Allonne, France) for *S. enterica*, and *E. coli* 0157:H7, and in 50 mL of TSB enriched with 6 g/L of yeast extract (TSBYE) for *L. monocytogenes*, followed by 24 h incubation at 37 °C. The cultures were centrifuged at 8900× *g* for 10 min, and the pellets were resuspended in a sterile Saline Solution (SS; 8.5 g/L NaCl, VWR, Radnor, PA, USA). At that point, all the strains were combined to obtain the cocktail. After that, a verification of the final population was made by plating in Xylose Lysine Desoxycholate Agar (XLD, Biokar, Allonne, France) for *S. enterica*, in MacConkey Sorbitol Agar (CT-SMAC Agar, Biokar, Allonne, France) with selective supplement for *E. coli* O157:H7, and in Palcam agar (Biokar, Allonne, France) with selective supplement for *L. monocytogenes*.

### 2.2. Sample Preparation

Apples, strawberries, and beetroots were purchased from a local supermarket. Before starting the experiments, the peduncle of strawberries was removed, apples were washed with tap water and dried, and beetroot was washed and disinfected with sodium hypochlorite (10%, *w*/*w*) with agitation at 500 rpm for 5 min. After that time, the beetroot was rinsed with tap water.

### 2.3. Experimental Design

**Figure 1:**
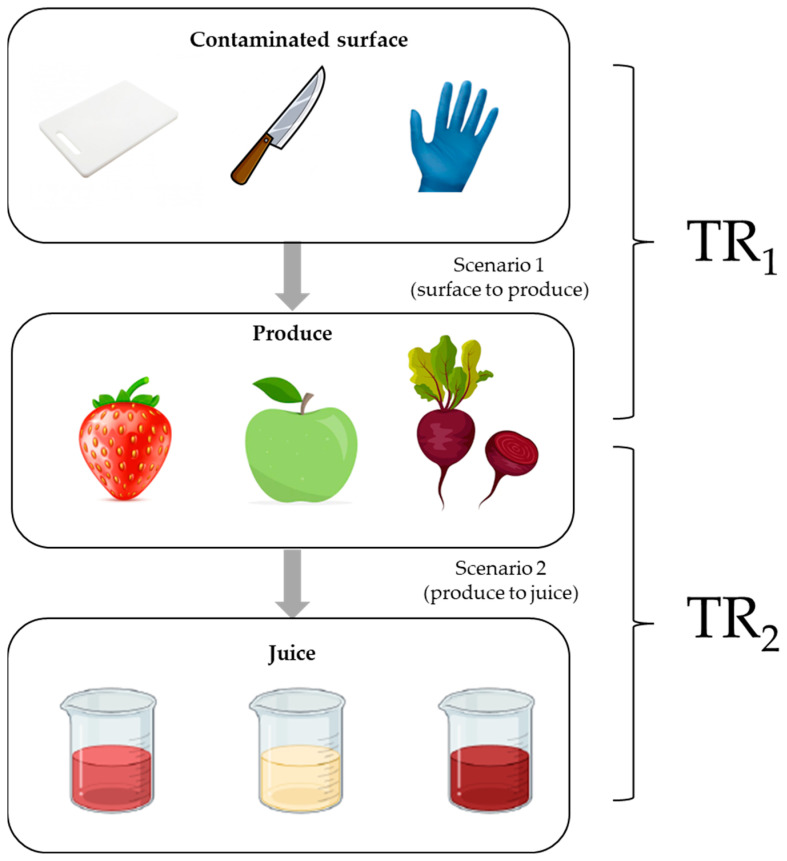
A schematic representation of the experimental design of the transfer assay.

**Figure 2:**
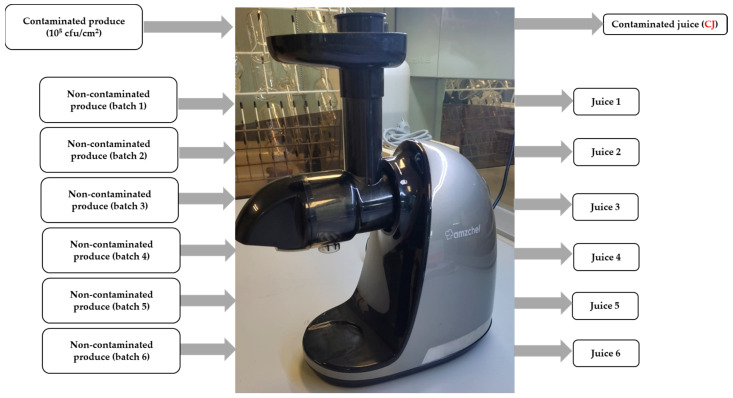
A schematic representation of the experimental design of the transfer of foodborne pathogens from contaminated produce to subsequent batches of juice. CJ: Contaminated juice obtained after processing contaminated produce.

The experiment consisted of two parts: (i) evaluating the transfer of foodborne pathogens from different surfaces (plastic cutting board, steel knife, and nitrile gloves) to the final processed juice; and (ii) assessing the transfer of foodborne pathogens from a first batch of contaminated fruit or vegetable to subsequent batches of juice prepared with non-contaminated produce using the same equipment. Schematic diagrams for each part are presented in Figure 1 and Figure 2, respectively. The following sections describe each part in detail.

#### 2.3.1. Transfer of Foodborne Pathogens from Surfaces to Juice

The transfer of foodborne pathogens from three artificially inoculated surfaces (cutting board, knife, and gloves) to juice was evaluated. Prior to inoculation, the apples and beetroots were cut into wedges to expose the flesh. Each fruit or vegetable was cut into 10 pieces (5 mm thick and 1.4 cm in diameter), providing a total contact area of 15.4 cm^2^.

Then, each surface was contaminated to obtain an initial population of approximately 10^6^ cfu/cm^2^ for each microorganism. To achieve this concentration, two microbial cocktails were prepared. The first cocktail, at 10^8^ cfu/mL, was used to contaminate the cutting board (100 cm^2^) and gloves (30 cm^2^) by spreading 1.0 and 0.3 mL, respectively. The second cocktail, at 10^9^ cfu/mL, was used to contaminate the knife by immersing it in the suspension for 1 min.

The transfer rate (TR) was assessed from two scenarios: (1) surface to the produce (TR_1_), and (2) produce to the juice (TR_2_). For the surface-to-produce scenario, after surface contamination, the fruits or vegetable pieces were contacted for 1 min. Then the fruits or vegetable pieces were placed in a blender bag (Interscience, Bag system, Saint-Nom-la Bretèche, France) containing 5 mL of Buffered Peptone Water (BPW; Biokar, Allone, France). The samples were homogenized in a blender (Interscience, Minimix, Saint-Nom-la Bretèche, France) for 90 s, followed by plating onto selective media for each microorganism.

Regarding the produce-to-juice scenario, the contaminated pieces of fruits and vegetables were combined with non-contaminated produce to obtain a total weight of approximately 100 g. This mixture was then processed using a cold press machine (Amzchef, No.: ZM1501, Xiaolan Town, Zhongshan, China) to determine pathogen transfer into the juice. The populations of *S. enterica*, *E. coli* O157:H7, and *L. monoctogenes* were determined by serial dilution in Saline Peptone (SP; 8.5 g/L of NaCl and 1 g/L of peptone) and plating onto selective media for each microorganism.

The results obtained for the transfer from surface to produce and from produce to juice were expressed as a TR in percentage, calculated using Equations (1) and (2), according to Pérez-Rodríguez et al. [24]:

(1) $$\%TR1=\frac{cfu\;on\;recipient\;surface}{cfu\;on\;donor\;produce}\;\cdot \;100$$

where the enumerator represents the number of cells (cfu) on the recipient surface, and in the denominator, the number of cells (cfu) in the donor produce.

(2) $$\%TR2=\frac{cfu\;on\;recipient\;produce}{cfu\;in\;donor\;juice}\;\cdot \;100$$

where the enumerator represents the number of cells (cfu) on recipient produce, and in the denominator, the number of cells (cfu) in the donor juices.

#### 2.3.2. Transfer of Foodborne Pathogens from Contaminated Fruit or Vegetable to Subsequent Batches of Juice

In this section, the transfer of bacterial strains from fruits and vegetables to juice and their potential carryover into successive juice batches prepared with non-contaminated produce were evaluated.

One hour before starting the experiments, the fruits and vegetables were removed from storage at 4 °C and kept at room temperature. For each product, eleven portions were prepared and marked with a surface area of 1.4 × 1.4 cm (1.96 cm^2^), which was then artificially inoculated with 50 µL of a microbial cocktail at approximately 10^6^ cfu/mL, yielding an initial load of 10^5^ cfu/cm^2^. Especially for strawberries, each unit represents a portion; in the case of apples and beetroots, a portion represents a wedge. All the portions were left at room temperature until the inoculum was fully dried. To determine the initial microbial load on the fruit surface, six contaminated portions were taken, and two portions were used per replicate (total inoculated area: 3.92 cm^2^). Then, the five inoculated portions were mixed with non-contaminated produce to obtain a total weight of approximately 100 g and processed using a cold press machine to obtain juice. After obtaining contaminated juice, six additional batches of non-contaminated produce were processed to obtain juice.

The microbial load of each pathogen in the juice was obtained by serially diluting and plating the adequate dilutions onto selective media appropriate to each microorganism. Incubation was carried out at 37 °C for 24 h for *S. enterica* and *E. coli* O157:H7 and 48 h for *L. monocytogenes*. Additionally, the pH and soluble solids content (SSC) of the last processed juice were measured. The measured values were for pH 4.14 ± 0.59, 4.02 ± 0.16, and 6.10 ± 0.09, and for SSC, 6.50 ± 0.80, 13.40 ± 1.20, and 9.30 ± 2.90 °Brix, for the strawberry, apple, and beetroot juices, respectively.

### 2.4. Data Analysis

All the data obtained was processed using the Microsoft 365 Excel software version 2506. For the transfer from surfaces to juice assay, data were first expressed as total cfu, and transfer ratios (TR_1_ and TR_2_) were calculated according to Equations (1) and (2). Subsequent statistical analysis by applying an analysis of variance (ANOVA) with the Tukey test (*p* < 0.05) determined significant differences in TR values for each microorganism across different matrices. All the analyses were performed using the JMP 17 Pro statistical software.

For the transfer of pathogens across different juice batches, the data was first log-transformed and plotted against the number of processed juices. Statistical differences across treatments were then assessed using ANOVA, followed by a Tukey test (*p* < 0.05). The experimental data obtained from the transfer curves (*n* = 6) were fitted to two mathematical models: Weibull plus tail and biphasic models. The kinetic parameters of microbial transfer were analyzed using the GInaFiT Excel add-in [25]. To assess how well the models fit the data, two parameters were considered: the adjusted R^2^ values (R^2^-_adj_), which indicate the model’s goodness of fit while accounting for the number of variables, and the Root Mean Square deviation (RMSE), which represents the average discrepancy between the observed and predicted values. The Weibull plus tail model equation [26] is given by the following:

(3) $$\log_{10}\left(\frac{N}{N_{0}}\right)=\;-{\left(\frac{d}{\delta }\right)}^{p}$$

where *d* represents the number of juices, *δ* (scale parameter) indicates the number of processed juices for the first decimal reduction, and *p* (dimensionless) is the shape parameter that determines concavity: *p* > 1 (concave down) and *p* < 1 (concave up), and biphasic model assumes a heterogeneous population with two subpopulations: one more sensitive (initial rapid decline) and one more resistant (tailing effect). The model equation is as follows:

(4) $$\log_{10}\left(\frac{N}{N_{0}}\right)\;=\log_{10}\left(fe^{-k_{max1}d}\;+\;\left(1\;-\;f\right)\;e^{-k_{max2}d}\right)$$

where *f* represents the fraction of the initial major subpopulation, and *k_max_*_1_ and *k_max_*_2_ are the transfer rate constants for the more sensitive and resistant subpopulations, respectively.

## 3. Results

### 3.1. Transfer of Foodborne Pathogens from Surfaces to Juice

The results of *S. enterica*, *E. coli* O157:H7, and *L. monocytogenes* transfer from the evaluated surfaces (cutting board, knife, and gloves) to produce (strawberry, apple, and beetroot, TR_1_) and from produce to its respective juice (TR_2_) are shown in Table 1 and Table 2.

The data revealed a similar pattern across the three pathogens evaluated (*Salmonella*, *E. coli* O157:H7, and *L. monocytogenes*) from surfaces to produce (TR_1_, Table 1). Beetroot exhibited the highest transfer rates from all the tested surfaces and microorganisms. For instance, the highest transfer rate was observed with *S. enterica* from the cutting board to beetroot (70.69 ± 23.58%), and with most values ranging between 48.85 ± 21.66% (*L. monocytogenes* from cutting board and gloves) to 69.09 ± 32.29% (*E. coli* O157:H7 from cutting board) across the rest of microorganisms and surfaces. In contrast, the knife showed the lowest transfer rates, such as 1.27 ± 1.35% for *L. monocytogenes* in strawberry. Overall, the strawberry was the least favorable matrix for pathogens transfer, particularly for *L. monocytogenes*, where the transfer rates ranged from 0.04 ± 0.05% (gloves) to 2.03 ± 4.36% (cutting board).

Table 2 highlights the highest transfer rates (TR_2_) observed from the contaminated apples via gloves to juice for *S. enterica* (31.42 ± 50.90%) and from the beetroot contaminated via gloves to juice for *L. monocytogenes* (17.01 ± 28.23%), although these differences were not statistically significant among the matrices and surfaces. A significant difference was only observed for *L. monocytogenes* in the apple juice contaminated from the cutting board (10.45 ± 8.80%). In contrast, the strawberries exhibited the lowest transfer, especially for *L. monocytogenes* (0.08 ± 0.06% from the cutting board and 0.20 ± 0.34% from the knife). Among surfaces, the knives consistently showed the lowest transfer rates regardless of the evaluated fruit and pathogen, while the gloves presented a higher variability. For example, a high transfer rate to juice was observed for *S. enterica* in the apple (31.42 ± 50.90%) originally contaminated from the glove, but a low transfer rate for *E. coli* O157:H7 in the beetroot (1.24 ± 0.49%) under the same scenario.

### 3.2. Transfer of Foodborne Pathogens from Juice Elaborated with Contaminated Produce to Subsequent Batches of Juice

**Figure 3:**
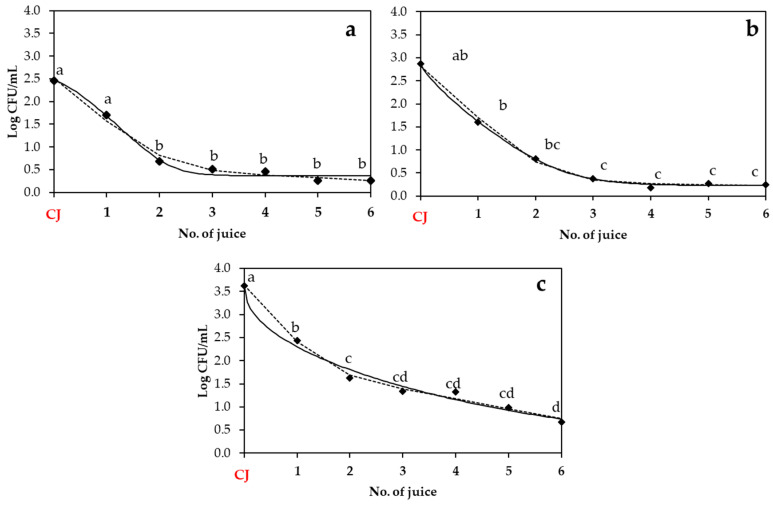
Population of *S. enterica* in strawberry (**a**), apple (**b**), and beetroot (**c**) juices obtained after elaboration of a contaminated juice (CJ) with contaminated produce. Experimental data points (◆) are compared with two mathematical models: the Weibull plus tail model (solid line) and the biphasic model (dashed line). Different lowercase letters indicate statistically significant differences (*p* < 0.05) among No. of juice within each fruit type, as determined by ANOVA followed by Tukey’s HSD test. CJ: Contaminated juice obtained after processing contaminated produce.

**Figure 4:**
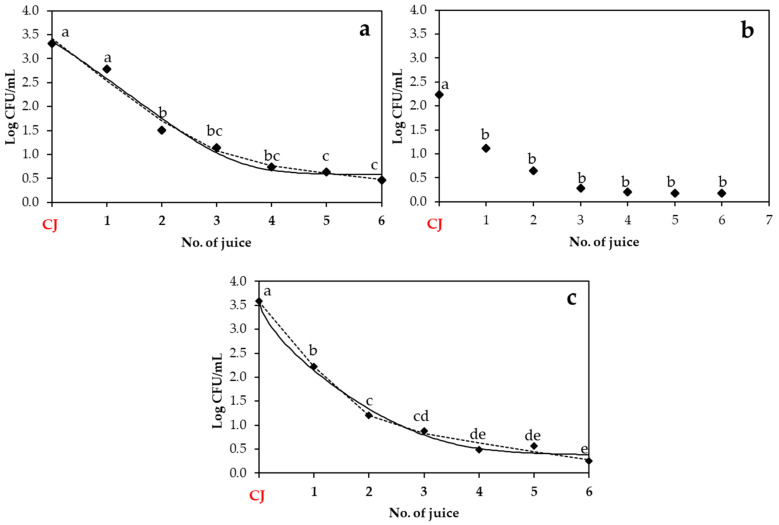
Population of *E. coli* O157:H7 in strawberry (**a**), apple (**b**), and beetroot (**c**) juices obtained after elaboration of a contaminated juice (CJ) with contaminated produce. Experimental data points (◆) are compared with two mathematical models: the Weibull plus tail model (solid line) and the biphasic model (dashed line). Different lowercase letters indicate statistically significant differences (*p* < 0.05) among No. of juice within each fruit type, as determined by ANOVA followed by Tukey’s HSD test. CJ: Contaminated juice obtained after processing contaminated produce.

**Figure 5:**
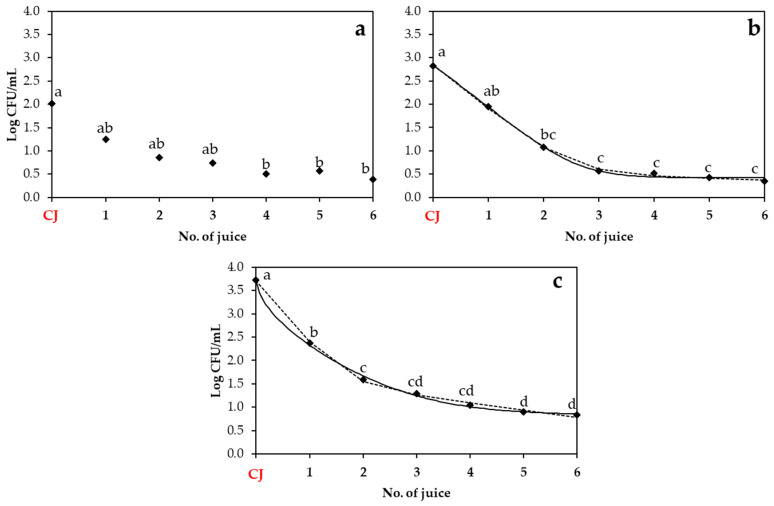
Population of *L. monocytogenes* in strawberry (**a**), apple (**b**), and beetroot (**c**) juices obtained after elaboration of a contaminated juice (CJ) with contaminated produce. Experimental data points (◆) are compared with two mathematical models: the Weibull plus tail model (solid line) and the biphasic model (dashed line). Different lowercase letters indicate statistically significant differences (*p* < 0.05) among No. of juice within each fruit type, as determined by ANOVA followed by Tukey’s HSD test. CJ: Contaminated juice obtained after processing contaminated produce.

In this part, the transfer of S. enterica, *E. coli* O157:H7, and *L. monocytogenes* from the strawberry, apple, and beetroot juices elaborated with contaminated produce (Figure 3, Figure 4 and Figure 5) to subsequent batches of juice was evaluated and adjusted to two mathematical models: the Weibull plus tail and the biphasic models. The adequacy of the models was assessed using goodness-of-fit parameters (adjusted R^2^ and RMSE, as shown in Table 3), in addition to their corresponding kinetic parameters (Table 4).

Figure 3 shows the results of *S. enterica* transfer into strawberry, apple, and beetroot juices across different batches. A different reduction pattern in its population dynamics was observed. In strawberry juice (Figure 3a), a significant reduction in the population was observed between the contaminated juice (CJ) and the second non-contaminated juice batch, decreasing from 2.46 ± 0.57 to 0.69 ± 0.56 log cfu/mL. From the third juice onward, the population levels stabilized, maintaining over 0.3–0.5 log cfu/mL. A similar trend was observed with apple juice (Figure 3b), with an initial load in the contaminated juice of 2.87 ± 0.89 log cfu/mL, showing significant differences in its population after the third juice batch (0.38 ± 0.31 log cfu/mL), followed by a stabilization of the population. In contrast, beetroot juice (Figure 3c) showed a different trend, with *S. enterica* populations ranging from 3.62 ± 0.37 log cfu/mL in the contaminated juice, and a significant reduction was observed after processing the first juice (log cfu/mL). Regarding data model fitting, both the Weibull plus tail and biphasic models provided good adjustments across matrices, with R^2^-_adj_ ranging between 0.710 and 0.862, and RMSE values between 0.374 and 0.495 (Table 3). The kinetic parameters (Table 4) reflect the described trends: beetroot juice exhibited the most rapid initial decrease (lowest *δ*, 0.53 ± 0.26) and the highest *k_max_*_1_ (3.09 ± 0.63), while strawberry and apple had *δ* values of 1.18 ± 0.27 and 0.75 ± 0.25, and *k_max_*_1_ values of 2.28 ± 0.56 and 2.61 ± 0.44, respectively.

*E. coli* O157:H7 showed different transfer patterns across juice matrices. In the strawberry juice (Figure 4a), its population decreased significantly from 2.83 ± 0.98 log cfu/mL in the contaminated juice to 1.31 ± 0.74 log cfu/mL in the second juice, followed by a stabilization phase, reaching 0.42 ± 0.34 log cfu/mL in the sixth batch juice. In the apple juice (Figure 4b), a significant decrease occurred from the CJ (2.23 ± 1.16 log cfu/mL) to the first non-contaminated juice (1.12 ± 0.67 log cfu/mL); however, no further significant decrease was observed. In the beetroot juice (Figure 4c), an initial decline from 3.58 ± 0.46 to 2.22 ± 0.41 log cfu/mL was observed after the first subsequent non-contaminated juice batch, followed by a continuous and gradual decrease across all batches. Model fitting varied among matrices (Table 3). In the strawberry juice, both models yielded similar fits (R^2^-_adj_ = 0.820 for biphasic, 0.817 for Weibull plus tail; and RMSE approximately 0.48 for both models). In the apple juice, data could not be properly fitted by the tested models, while in the beetroot juice, the biphasic model provided the best fit (R^2^-_adj_ = 0.922; RMSE = 0.324). Among matrices, beetroot showed the most consistent reduction, with the lowest δ (0.56 ± 0.16) and highest *k_max_*_1_ (3.20 ± 0.39), compared to strawberry (*δ* = 1.26 ± 0.30; *k_max_*_1_ = 2.08 ± 0.39) (Table 4).

*L. monocytogenes* also exhibited distinct transfer patterns across the different juice types. In the strawberry juice (Figure 5a), the bacterial population decreased from 2.01 ± 1.22 log cfu/mL in the initially contaminated juice to 0.51 ± 0.51 log cfu/mL by the fourth juice. Thereafter, the population remained stable with no significant changes throughout the subsequent juices. In the apple juice (Figure 5b), a fast initial decline was observed from the CJ (2.83 ± 0.58 log cfu/mL) to the second juice (1.07 ± 0.84 log cfu/mL), followed by a stabilization of population with no significant changes thereafter. In the beetroot juice (Figure 5c), a continuous significant decrease was observed across juice batches: from 3.72 ± 0.29 log cfu/mL in the CJ to populations around 1–2 log cfu/mL across juice batches. As regards the model fitting, the beetroot juice was the matrix that demonstrated the best fit to both models tested (R^2^-_adj_ at approximately 0.90 and RMSE at 0.318). In contrast, the apple juice showed the worst model fit (approximately 0.65 for R^2^-_adj_ and 0.63 for RMSE), while the data from the strawberry juice could not be fitted to the tested models. A comparison of kinetic parameters shows that the beetroot juice resulted in the most effective microbial reduction, with the lowest *δ* (0.55 ± 0.18) and the highest *k_max_*_1_ (3.20 ± 0.46), indicating faster transfer reduction. In contrast, the apple juice showed a higher δ value (1.10 ± 0.38) and slower *k_max_*_1_ (2.20 ± 0.58) compared to beetroot.

## 4. Discussion

The growing demand for unpasteurized juices, valued for their nutritional benefits, presents a significant food safety concern. Additionally, the introduction of new fruits and vegetables into these juices may increase the risk of foodborne diseases, especially when conditions favor pathogen survival. Factors like high pH can further enhance microbial retention, increasing the risk of foodborne disease. This study is one of the first to not only assess pathogen transfer from three different surfaces to two different fruits and one vegetable but also to evaluate their transfer from a first contaminated batch of juice to subsequent non-contaminated juice batches, mimicking unpasteurized commercial juices. This novel approach provides critical insights into microbial risks beyond initial contamination, highlighting potential food safety challenges for fruit juice manufacturers.

In general, a high variability in the transfer ratios was observed. This can be attributed to the inherent heterogeneity of whole fruits and vegetables. Variations in contaminant distribution across surfaces, as well as differences in morphological and compositional traits (such as water content, natural wax layers, surface roughness, and skin thickness), can influence microbial behavior and adhesion [27].

Similar transfer patterns were observed for *S. enterica* and *E. coli* O157:H7 when evaluating their transfer from contaminated surfaces to produce. Both pathogens showed the highest transfer ratios observed from both the cutting board (ranging from 23.58 to 70.69%) and gloves (ranging from 14.17 to 70.61%). In contrast, the knife consistently showed the lowest transfer rates, with values ranging from 4.17 to 7.69% across the three different matrices. These differences in transfer rates could be explained by the inherent porosity and hydrophobicity of plastic (e.g., cutting boards) and nitrile (e.g., gloves) surfaces, which are known to influence pathogen transfer [28,29]. However, these findings differ from those reported by Ravishankar et al. [30], who observed a transfer rate of 45.62% for *S. enterica* from a knife to lettuce. Similarly, Jensen et al. [31] reported transfer rates between 80 and 98% when *Salmonella* spp. and *E. coli* O157:H7 were transferred from plastic or stainless-steel surfaces to produce such as carrot, celery, lettuce, and watermelon. These discrepancies may be attributed to differences in experimental conditions, surface properties, or moisture presence, all of which can significantly influence bacterial adhesion and transfer efficiency. In contrast, *L. monocytogenes* demonstrated a distinct pattern in the present study, generally showing lower transfer rates across all the surfaces compared to *S. enterica* and *E. coli* O157:H7. Notably, strawberry was the matrix in which the lowest transfer rates were observed for *L. monocytogenes*, ranging from 0.04 to 2.03%, suggesting a greater sensitivity of this pathogen to this matrix.

Beyond surface-to-produce transfer, this study also evaluates the transfer of pathogens from produce to juice. *S. enterica* exhibited the highest transfer rate (31.42 ± 50.90%), particularly from apple contaminated through gloves to juice. Similarly, *L. monocytogenes* showed a high transfer ratio from produce contaminated via gloves, but in this case, beetroot was the matrix with a higher transfer rate (17.01 ± 28.23%). When the initial donor surface was a cutting board, *S. enterica* showed significant differences in the transfer rate of apple and strawberry, with a higher transfer rate in apple, and the same pattern was observed in *L. monocytogenes*. By contrast, *E. coli* O157:H7 displayed a different transfer pattern, with no significant differences between matrices regardless of the initial donor surface evaluated. Although there are no specific studies on the transfer of pathogens from contaminated fruits or vegetables to their corresponding juice, previous research has shown that once pathogens are introduced to juice, they are strongly influenced by the physicochemical properties of the juice matrix, including acidity, polyphenol content, and buffering capacity, which can either promote or inhibit microbial transfer [20,21,22].

Focusing on differences among matrices, our study found that beetroot exhibited the highest pathogen transfer rates from donor surface to fruit across all the tested pathogens, while strawberries showed the lowest transfer rates, particularly in the case of *L. monocytogenes* when the donor surface was a knife. This variability can be attributed to both donor and recipient characteristics; however, the literature suggests that the donor surface generally plays a more decisive role in the transfer process. Nonetheless, the intrinsic properties of the recipient (such as fruit or vegetable) also contributed significantly. The tested matrices varied in porosity, structure, and pH; factors known to influence pathogen adhesion. Generally, high-moisture, low-pH produce tends to retain bacteria more effectively [19,31,32]. Nevertheless, the physiological traits of the pathogen must also be considered. For instance, *L. monocytogenes* grows optimally in neutral to alkaline environments and is less adapted to acidic conditions such as those found in strawberries. This mismatch between the pathogen’s growth preference and the fruit’s intrinsic pH may partially explain the reduced transfer and survival rates observed in this matrix [33,34].

This study also evaluated the transfer of pathogens across consecutive batches of non-contaminated juice following an initial contamination. A rapid decline in microbial load was observed immediately after processing the first contaminated batch, despite initial inoculation levels of approximately 5 log cfu/cm^2^ on the surfaces of produce. Specifically, microbial populations dropped to around 2–3 log cfu/mL in the strawberry and apple juices, and to 3–4 log cfu/mL in the beetroot juice. The immediate reduction appears to be strongly influenced by matrix-specific effects. Although beetroot had a higher pH than strawberry and apple (6.10 ± 0.09 for beetroot, 4.14 ± 0.59 for strawberry, and 4.02 ± 0.16 for apple), it showed the most rapid reduction (indicated by δ and *k*_max1_ values) in pathogen levels. This effect may be attributed to beetroot’s high anthocyanin content, which has previously been reported to exert inhibitory effects on *Salmonella* and *E. coli* [35,36].

To further investigate the observed trends, two mathematical models were applied to evaluate the transfer dynamics of *S. enterica*, *E. coli* O157:H7, and *L. monocytogenes* across the different juice matrices. The number of juice batches required to achieve the first decimal reduction varied by matrix, with strawberry requiring the most, followed by apple and beetroot. This trend was reflected in the model parameters: the strawberry juice exhibited the highest δ values (1.18–1.26) and the lowest *k*_max1_ values (2.08–2.28), suggesting that more juice batches were required to achieve the first decimal reduction. In contrast, the beetroot juice exhibited the slowest bacterial decrease, with lower δ values (0.53–0.56) and higher *k*_max1_ values (3.09–3.20). These results suggest that beetroot’s physicochemical profile (characterized by the presence of betacyanin’s) may contribute to enhanced antimicrobial effects [37]. Compared with beetroot and strawberry, apple juice demonstrated intermediate behavior. For *S. enterica* and *L. monocytogenes*, δ values ranged from 0.75 to 1.10, and *k*_max1_ values were between 2.20 and 2.61. This moderate decline in the microbial population may be linked to the presence of malic acid, which, as a known antimicrobial compound, can contribute to bacterial stress by lowering intracellular pH and interfering with key metabolic functions [38].

The European Food Safety Authority (EFSA) reported 11 outbreaks linked to *Salmonella* spp. in the category of vegetables and juices in 2021, with *S.* Enteritidis identified as the most prevalent serovar [39]. These findings reinforce concerns about the role of fresh juices as potential vehicles for foodborne pathogens. Furthermore, the Spanish Agency for Food Safety and Nutrition (AESAN) highlighted in a 2022 scientific committee report that contamination of fruit pulp with pathogens is possible and that such contamination can occur during the cutting and handling of fruit peels and skins, posing a potential food safety risk [40]. The results of the present study reinforce these concerns, demonstrating that pathogens present in fruits and vegetables can be transferred to juice during preparation, particularly in the absence of pasteurization or other pathogen elimination processes. Moreover, the findings suggest that gloves may pose a greater contamination risk when handling fruits or vegetables, as previously reported in studies on cross-contamination in food processing environments. However, the role of cutting boards should not be underestimated, as previous research has shown that plastic and other porous materials can retain microorganisms and subsequently facilitate their transfer to food [41,42].

## 5. Conclusions

Given that unpasteurized juices do not undergo processes to eliminate pathogens, understanding microbial behavior during juice production is crucial for enhancing food safety in minimally processed products. This study emphasized the importance of evaluating pathogen transfer from contaminated surfaces (cutting boards, knives, and gloves) to produce and their corresponding juices. Additionally, assessing the transfer of pathogens from contaminated juice to subsequent non-contaminated juices is essential for identifying critical control points in juice processing. This study is the first to evaluate pathogen transfer at each step of unpasteurized strawberry, apple, and beetroot juice production. The findings reveal significant food safety risks in unpasteurized juice production, particularly due to high pathogen transfer rates from processing surfaces and the prolonged survival of microorganisms in strawberry, apple, and beetroot juices. Cutting boards and gloves exhibited higher contamination potential, reinforcing the need for enhanced cleaning and disinfection protocols. Additionally, in the beetroot juice, all the pathogens displayed higher transfer rates, suggesting that this matrix may be more favorable for their transfer. To minimize contamination risks, stricter hygiene protocols must be implemented, emphasizing enhanced cleaning and disinfection of processing equipment and utensils. Given the transfer of *S. enterica*, *E. coli* O157:H7, and *L. monocytogenes* in beetroot and apple juices, the industry should explore alternative preservation methods, such as high-pressure processing or antimicrobial compounds. A multi-hurdle approach combining sanitation, optimized processing, and antimicrobial strategies will enhance food safety, prolong shelf life, and meet consumer demand for fresh, minimally processed juices without compromising microbiological safety.

## Figures and Tables

**Table 1:** Transfer rate (TR_1_, %) of *S. enterica*, *E. coli* O157:H7, and *L. monocytogenes* from cutting board, knife, and gloves to strawberry, apple, and beetroot.

Microorganism	Matrix	Cutting Board	Knife	Gloves
*S. enterica*	Strawberry	24.17 ± 17.31 Bx	6.24 ± 2.84 Ay	24.68 ± 3.54 Bx
	Apple	43.33 ± 14.85 ABx	6.88 ± 6.98 Ay	30.29 ± 31.91 Bxy
	Beetroot	70.69 ± 23.58 Ax	7.17 ± 6.17 Ay	70.61 ± 23.51 Ax
*E. coli* O157:H7	Strawberry	23.58 ± 12.90 Ax	4.17 ± 2.92 Ay	14.17 ± 3.74 Bxy
	Apple	58.07 ± 59.40 Ax	7.48 ± 3.58 Ax	28.17 ± 19.97 Bx
	Beetroot	69.08 ± 32.29 Ax	7.69 ± 2.50 Ay	57.98 ± 16.17 Ax
*L. monocytogenes*	Strawberry	2.03 ± 4.36 Bx	1.27 ± 1.35 Bx	0.04 ± 0.05 Bx
	Apple	16.21 ± 13.68 Bx	5.64 ± 4.04 ABxy	2.80 ± 1.67 By
	Beetroot	48.85 ± 21.66 Ax	7.87 ± 5.33 Ay	48.85 ± 21.66 Ax

The values are expressed as mean ± standard deviation of six replicates (*n* = 6). Different capital letters (A, B) mean significant differences between matrices for each bacterium and surface, and different lowercase letters (x, y) mean significant differences between surfaces for each bacterium and matrix according to the Tukey test (*p* < 0.05).

**Table 2:** Transfer rate (TR_2_, %) of *S. enterica*, *E. coli* O157:H7, and *L. monocytogenes* from strawberry, apple, and beetroot contaminated through contact with cutting board, knife, and gloves, to juice.

Microorganism	Matrix	Cutting Board	Knife	Gloves
*S. enterica*	Strawberry	2.51 ± 2.42 Bx	3.86 ± 5.13 Ax	5.22 ± 1.89 Ax
	Apple	7.48 ± 4.32 Ax	1.75 ± 1.73 Ax	31.42 ± 50.90 Ax
	Beetroot	3.80 ± 1.85 ABx	2.33 ± 1.55 Ax	2.04 ± 1.35 Ax
*E. coli* O157:H7	Strawberry	4.16 ± 4.55 Ax	2.32 ± 2.65 Ax	8.88 ± 5.97 Ax
	Apple	9.24 ± 7.83 Ax	1.03 ± 0.71 Ax	8.18 ± 8.49 Ax
	Beetroot	4.29 ± 0.56 Ax	2.03 ± 1.17 Ay	1.24 ± 0.49 Ay
*L. monocytogenes*	Strawberry	0.08 ± 0.06 Bx	0.20 ± 0.34 Bx	4.50 ± 8.98 Ax
	Apple	10.45 ± 8.80 Ax	0.55 ± 0.25 ABy	4.77 ± 2.81 Axy
	Beetroot	4.56 ± 1.54 ABx	5.65 ± 6.08 Ax	17.01 ± 28.23 Ax

The values are expressed as mean ± standard deviation of six replicates (*n* = 6). Different capital letters (A, B) mean significant differences between matrices for each bacterium and surface, and different lowercase letters (x, y) mean significant differences between surfaces for each bacterium and matrix according to the Tukey test (*p* < 0.05).

**Table 3:** Statistical index of the goodness of fit for the Weibull plus tail and biphasic models describing the transfer of *S. enterica*, *E. coli* O157:H7, and *L. monocytogenes* after processing a contaminated juice and six consecutive uncontaminated juices.

Microorganism	Matrix	Weibull Plus Tail Model	Biphasic Model
*S. enterica*	Strawberry	R^2^-_adj_ = 0.709 RMSE = 0.495	R^2^-_adj_ = 0.710 RMSE = 0.495
	Apple	R^2^-_adj_ = 0.803 RMSE = 0.459	R^2^-_adj_ = 0.801 RMSE = 0.462
	Beetroot	R^2^-_adj_ = 0.852 RMSE = 0.387	R^2^-_adj_ = 0.862 RMSE = 0.374
*E. coli* 0157:H7	Strawberry	R^2^-_adj_ = 0.817 RMSE = 0.486	R^2^-_adj_ = 0.820 RMSE = 0.483
	Apple	-	-
	Beetroot	R^2^-_adj_ = 0.917 RMSE = 0.333	R^2^-_adj_ = 0.922 RMSE = 0.324
*L. monocytogenes*	Strawberry	-	-
	Apple	R^2^-_adj_ = 0.649 RMSE = 0.639	R^2^-_adj_ = 0.649 RMSE = 0.638
	Beetroot	R^2^-_adj_ = 0.904 RMSE = 0.318	R^2^-_adj_ = 0.904 RMSE = 0.318

R^2^-_adj_ represents the adjusted coefficient of determination, indicating the goodness of fit of the model to the data. RMSE corresponds to the Root Mean Square Error. The dashes (-) indicate that the models were not adjusted.

**Table 4:** Kinetic parameters obtained by fitting with the Weibull and biphasic models to the transfer of *S. enterica*, *E. coli* O157:H7, and *L. monocytogenes* during the processing of consecutive juice batches.

Microorganism	Matrix	Weibull Plus Tail Model		Biphasic Model		
		*δ*	*p*	*f*	*k_max_*_1_	*k_max_*_2_
*S. enterica*	Strawberry	1.18 ± 0.27	1.32 ± 0.51	0.99 ± 0.01	2.28 ± 0.56	0.13 ± 0.24
	Apple	0.75 ± 0.25	0.80 ± 0.25	0.99 ± 0.00	2.61 ± 0.44	0.05 ± 0.22
	Beetroot	0.53 ± 0.26	0.46 ± 0.13	0.98 ± 0.02	3.09 ± 0.63	0.49 ± 0.14
*E. coli* O157:H7	Strawberry	1.26 ± 0.30	1.06 ± 0.28	0.99 ± 0.00	2.08 ± 0.39	0.27 ± 0.34
	Apple	-	-	-	-	-
	Beetroot	0.56 ± 0.16	0.66 ± 0.12	0.99 ± 0.00	3.20 ± 0.39	0.41 ± 0.13
*L. monocytogenes*	Strawberry	-	-	-	-	-
	Apple	1.10 ± 0.38	1.03 ± 0.45	0.99 ± 0.01	2.20 ± 0.58	0.09 ± 0.35
	Beetroot	0.55 ± 0.18	0.58 ± 0.12	0.99 ± 0.01	3.20 ± 0.46	0.37 ± 0.12

Values are presented as mean ± standard deviation. *δ* presents the scale parameter; *p* is the shape parameter; *f* is the fraction of the bacterial population; *k_max_*_1_ and *k_max_*_2_ are the transfer rate constants. Dashes (-) indicate cases where no suitable fit was obtained.

## Data Availability

The data presented in this study are available upon request from the corresponding author.
